# A Sensitive Aptamer Fluorescence Anisotropy Sensor for Cd^2+^ Using Affinity-Enhanced Aptamers with Phosphorothioate Modification

**DOI:** 10.3390/bios12100887

**Published:** 2022-10-17

**Authors:** Hao Yu, Qiang Zhao

**Affiliations:** 1 State Key Laboratory of Environmental Chemistry and Ecotoxicology, Research Center for Eco-Environmental Sciences, Chinese Academy of Sciences, Beijing 100085, China; 2 University of Chinese Academy of Sciences, Beijing 100049, China; 3 School of Environment, Hangzhou Institute for Advanced Study, University of Chinese Academy of Sciences, Hangzhou 310000, China

**Keywords:** cadmium ions, aptamer, fluorescence, biosensors, fluorescence polarization

## Abstract

Rapid and sensitive detection of heavy metal cadmium ions (Cd^2+^) is of great significance to food safety and environmental monitoring, as Cd^2+^ contamination and exposure cause serious health risk. In this study we demonstrated an aptamer-based fluorescence anisotropy (FA) sensor for Cd^2+^ with a single tetramethylrhodamine (TMR)-labeled 15-mer Cd^2+^ binding aptamer (CBA15), integrating the strengths of aptamers as affinity recognition elements for preparation, stability, and modification, and the advantages of FA for signaling in terms of sensitivity, simplicity, reproducibility, and high throughput. In this sensor, the Cd^2+^-binding-induced aptamer structure change provoked significant alteration of FA responses. To acquire better sensing performance, we further introduced single phosphorothioate (PS) modification of CBA15 at a specific phosphate backbone position, to enhance aptamer affinity by possible strong interaction between sulfur and Cd^2+^. The aptamer with PS modification at the third guanine (G) nucleotide (CBA15-G3S) had four times higher affinity than CBA15. Using as an aptamer probe CBA15-G3S with a TMR label at the 12th T, we achieved sensitive selective FA detection of Cd^2+^, with a detection limit of 6.1 nM Cd^2+^. This aptamer-based FA sensor works in a direct format for detection without need for labeling Cd^2+^, overcoming the limitations of traditional competitive immuno-FA assay using antibodies and fluorescently labeled Cd^2+^. This FA method enabled the detection of Cd^2+^ in real water samples, showing broad application potential.

## 1. Introduction

Heavy metal pollutant cadmium ion (Cd^2+^) is released into the environment through natural and anthropogenic activities such as industrial emissions, agricultural fertilization, metallurgy, and mining [1,2]. Emissions of Cd^2+^ into the water, soil, and air can lead to serious pollution and deposition of fauna and flora, and Cd^2+^ can be eventually accumulated in the human body through the food chain [1,2,3,4,5]. Cd^2+^ is highly toxic and causes adverse effects on human health, such as itai-itai disease, hypertension, and cancers [1,2,3,4]. The U.S. Environmental Protection Agency (EPA) sets the maximum concentration of Cd^2+^ as 5.0 μg/L in drinking water. As Cd^2+^ contamination and Cd^2+^ exposure are widespread, Cd^2+^ detection is important and necessary for food safety, environmental monitoring, and health risk assessment [6]. Conventional methods for Cd^2+^ detection include atomic absorption spectrometry, inductively coupled plasma atomic emission spectrometry, inductively coupled plasma mass spectrometry, etc. [6,7,8]. Despite high sensitivity and accuracy, certain limitations include expensive instruments, complicated sample pretreatment, and time-consuming operations, and the process is not suitable for rapid onsite monitoring of Cd^2+^. Therefore, there is an urgent need to develop simple, rapid and cost-effective methods to detect trace amounts of Cd^2+^. Biosensors for Cd^2+^ can meet these demands, and have attracted considerable attention [6,7].

Aptamers are single-stranded oligonucleotides that bind to targets with high specificity and affinity [9,10]. As new recognition elements in biosensors, aptamers show advantages over antibodies in terms of low cost, good thermal stability, facile production, easy modification with functional groups, and binding-induced conformation change. Aptamer-based sensors have enabled detection of various targets such as metal ions, small molecules, proteins, and cells, due to the appealing features of aptamers [11,12,13,14]. Since aptamers for Cd^2+^ were identified [15,16], aptamer-based sensors and assays for Cd^2+^ have become possible and have provided new approaches for detection of Cd^2+^, including electrochemistry, colorimetry, and fluorescent methods [15,16,17,18,19,20,21].

Fluorescence polarization (FP)/anisotropy (FA) is an attractive fluorescence technique due to its sensitivity, simplicity, and homogeneous and high-throughput analysis, and is often used in environmental monitoring, drug discovery, food analysis, and affinity-binding research [22,23,24,25,26,27]. As a ratiometric method, FA assay can eliminate the influence of fluorescence fluctuation and photobleaching, and shows high reproducibility [22,23,24]. Combining the advantages of aptamer and FP/FA analysis, the use of aptamers in FP/FA assays further improves applications of FA analysis, allowing development of versatile formats of FA assays for various targets [23,24,26,27]. Among the available aptamer-based FA assays, the FA assay using tetramethylrhodamine (TMR)-labeled aptamers is a simple non-competitive method, which relies on target-binding-induced change of TMR–nucleotide (e.g., TMR-G) interaction, without the need for fluorescent labeling of targets [27]. FP/FA methods for the detection of Cd^2+^ are limited and their development is challenging. The immunoantibody-based FP method is a competitive assay and requires the Cd^2+^ complex to be labeled with fluorophore and the antibody for Cd^2+^ chelate [28], with limitations encountered in the preparation of antibodies against Cd^2+^ and fluorescence tracers of Cd^2+^ [28,29].

In this paper, we report a simple aptamer FA sensor in a direct format for rapid sensitive detection of Cd^2+^ using a single TMR labeled 15-mer Cd^2+^ binding aptamer (CBA15). By screening FA responses of different labeling sites of TMR (3′ end, 5′ end, and internal T bases), we identified that the aptamer-labeled TMR on the 12th T base showed remarkable FA change upon Cd^2+^ binding. In addition, to improve assay sensitivity, a high-affinity aptamer is desirable. We further demonstrated a strategy of introduction a single phosphorothioate (PS) modification to the aptamer, by replacing one of the phosphate oxygen atoms with sulfur, to greatly enhance aptamer affinity by tightening the binding between Cd^2+^ and the aptamer, with strong interaction between Cd^2+^ and sulfur [30]. We found that when PS modification was introduced to the linker between the third nucleotide G and the fourth nucleotide G of CBA15 (CBA15-G3S), a stronger aptamer affinity to Cd^2+^ was obtained, with a K_d_ about 47 nM representing a more than four-fold improvement in affinity compared with CBA15 without PS modification. We employed CBA15-G3S with optimal TMR label at the 12th T (CBA15-G3S-T12-TMR) in the FA sensor, and achieved more sensitive FA detection of Cd^2+^ with a detection limit at the nM level. The aptamer FA sensor allowed detection of Cd^2+^ in tap water and lake water samples, showing applicability for analysis of Cd^2+^ in a complex sample matrix.

## 2. Materials and Methods

### 2.1. Chemical and Reagents

CdCl_2_ was ordered from J & K Chemicals (Beijing, China). Pb(Ac)_2_, Mn(Ac)_2_, NiCl_2_, CuCl_2_, and ZnSO_4_ were ordered from Sangon Biotech (Shanghai, China). HgCl_2_ was purchased from National Pharmaceutical Group. MgCl_2_ and NaCl were obtained from Sinopharm (Shanghai, China). All of the unlabeled DNA aptamers, DNA aptamers with PS modification, and DNA aptamers with single tetramethylrhodamine (TMR) labeled at different sites (3′ end, internal thymine bases (T), and 5′ end) were synthesized and purified by Sangon Biotech (Shanghai, China). The sequences are listed in Table 1. The binding buffer used in this experiment was prepared with ultrapure water (18.2 MΩ·cm) from Purelab Ultra Elga Labwater (Buckinghamshire, England). Other reagents used in this experiment were of analytical grade.

### 2.2. Isothermal Titration Calorimetry Measurement

Isothermal titration calorimetry (ITC) analysis was carried out at 25 °C using a MicroCal PEAQ-ITC (Malvern, Malvern, UK) to determine aptamer affinity. The binding buffer for ITC analysis contained 20 mM Tris-HCl (pH 7.5) and 20 mM NaCl. During ITC measurements, the reference power was set to 10 μcal/s and the stirring speed of the syringe was 750 rpm. Cd^2+^ solution (200 μM) from the injection syringe was titrated into the aptamer solution (20 μM) in a sample cell. After 60 s initial delay, the experiment began with the first 0.4 μL of Cd^2+^ solution and 19 successive 2.0 μL of Cd^2+^ solution every 100 s. The binding curves were obtained by integrating the heat pulse areas of each titration. Dissociation constants (K_d_s), enthalpy change (ΔH), and entropy change (TΔS) were obtained by fitting the one-site binding model with the packaged MicroCal PEAQ-ITC analysis software.

### 2.3. Fluorescence Anisotropy Measurement

Certain concentrations of Cd^2+^ were mixed with dye-labeled aptamers (20 nM) in the binding buffer of 20 mM Tris-HCl (pH 7.5) and 20 mM NaCl. After incubation of the sample solution for 10 min at 25 °C, unless otherwise stated, fluorescence anisotropy (FA) measurements were conducted on a Synergy^TM^ H1 microplate reader (BioTek, Highland Park, IL, USA) with excitation at 530 nm and emission at 590 nm. Duplicate samples were tested for detection of Cd^2+^, and after three measurements of the same sample solution, the average data were used.

### 2.4. Detection of Cd^2+^ in Complex Sample Matrix

Tap water and lake water (Beijing Olympic Forest Park, China) were filtered through a 0.22 μm membrane, and then the treated water samples were diluted 20-fold with the binding buffer. Different concentrations of Cd^2+^ spiked in the complex sample matrix were tested by the aptamer-based FA assay, using the procedure described above.

## 3. Results and Discussion

### 3.1. FA Sensor for Cd^2+^ Using TMR-Labeled Aptamers

**Figure 1:**
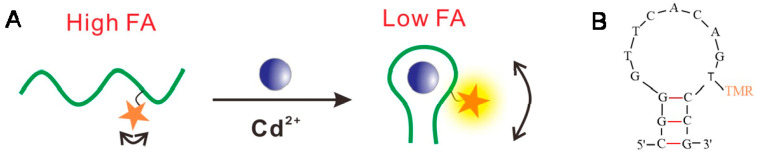
(**A**) Schematic diagram of fluorescence anisotropy sensor for Cd^2+^ using TMR-labeled aptamers. (**B**) The predicted secondary structure of the aptamer CBA15, with single TMR labeled on the 12th thymine base.

**Figure 2:**
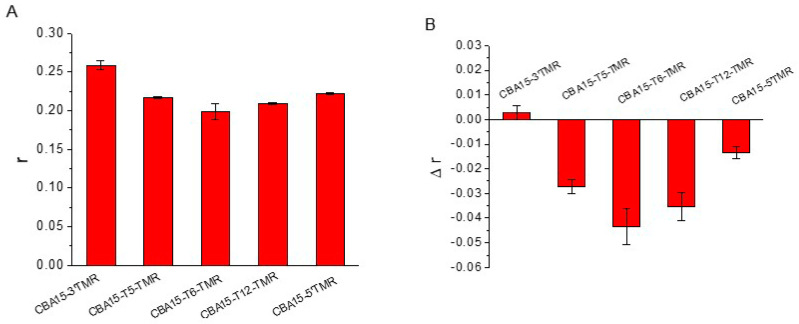
(**A**) FA responses of CBA15 with TMR labels at different sites (20 nM). (**B**) FA changes of TMR-labeled CBA15 caused by 500 nM Cd^2+^. Δr was obtained by subtracting the FA values of the blank sample from the FA values of 500 nM Cd^2+^.

Figure 1 shows the principle of the aptamer FA sensor for Cd^2+^ detection using TMR-labeled aptamers. It has been reported that TMR-G interaction may limit the local rotation of TMR and affect FA values [26,27,31]. When Cd^2+^ binds specifically to the aptamer, the conformation of the aptamer changes, which alters the TMR-G interaction and the FA signals of the TMR label. Therefore, the quantitative analysis of Cd^2+^ can be achieved by measuring changes in the FA signals of the TMR-labeled aptamer. In this method, an appropriate labeling site for TMR at the aptamer is required. To identify an appropriate position at the aptamer for TMR labeling, enabling the TMR-labeled aptamer to show sensitive FA response to Cd^2+^, we tested a series of sites of the 15-mer Cd^2+^-binding aptamer (CBA15), including 3′ end, internal thymine bases (5 T, 6 T, 12 T), and 5′ end (Table 1). CBA15 was truncated from a 21-mer aptamer sequence against Cd^2+^ [15,21]. We measured the FA responses of different TMR-labeled CBA15 to Cd^2+^ in the binding buffer containing 20 mM Tris-HCl (pH 7.5) and 20 mM NaCl. As shown in Figure 2A, most of these aptamer probes showed FA values higher than 0.200 when Cd^2+^ was absent, indicating possible TMR–nucleotide (e.g., G) interaction, causing high FA. In the presence of 500 nM Cd^2+^, the FA values of the aptamer probes decreased, except the probe CBA15-3′TMR (Figure 2B), suggesting that most of the TMR-labeled aptamers were FA-responsive to Cd^2+^. The FA decrease was due to Cd^2+^-binding-induced aptamer conformation change weakening the TMR-G interaction.

**Figure 3:**
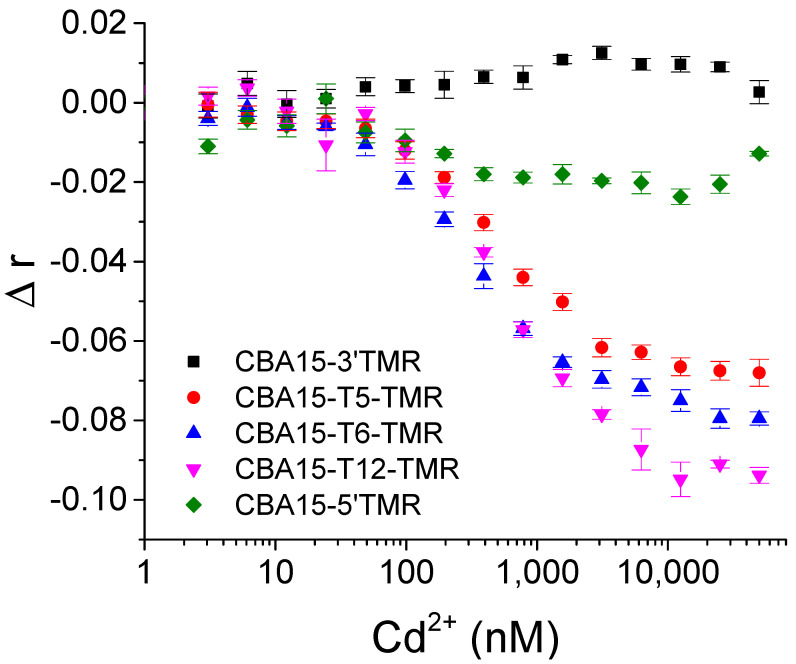
FA changes (Δr) of different TMR-labeled aptamers upon binding with various concentrations of Cd^2+^. Δr was obtained by subtracting the FA values of blank samples from the FA values of various concentrations of Cd^2+^.

We further tested the FA responses of different TMR-labeled aptamers upon addition of varying concentrations of Cd^2+^. As shown in Figure 3, CBA15-T5-TMR, CBA15-T6-TMR, and CBA15-T12-TMR showed significant FA decrease upon Cd^2+^ binding. CBA15-5′TMR exhibited slight FA decrease, while the CBA15-3′TMR showed small FA increase upon addition of Cd^2+^. When TMR was labeled on the internal 12th thymine (T) bases in the sequence, the corresponding aptamer probe CBA15-T12-TMR showed the largest FA signal change upon Cd^2+^ binding. Therefore, CBA15-T12-TMR was employed for FA detection of Cd^2+^.

### 3.2. Enhancing Aptamer Affinity with Phosphorothioate Modification

**Figure 4:**
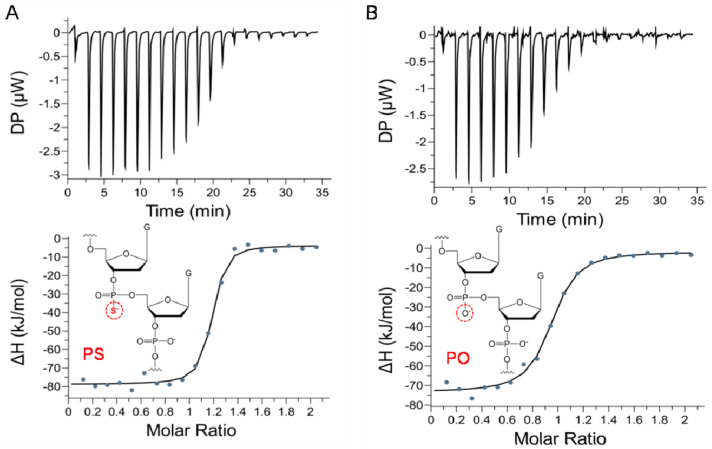
ITC analysis of aptamers (**A**) CBA15-G3S and (**B**) CBA15 with Cd^2+^. The top graph shows raw data for ITC titration, and the bottom graph displays the binding curve obtained by integrating the heats of each spike. The difference between PS modification and the phosphate (PO) group in the backbone of the aptamer is shown.

In order to improve the sensitivity of the aptamer FA sensor, aptamers with higher affinity are desirable. We attempted to introduce a single PS modification at a specific backbone site of the aptamer CBA15 (Table S1), to enhance affinity for the possible strong interaction between Cd^2+^ and the adjacent sulfur [30]. We determined the affinity of aptamers with PS modification at different labeling sites, by ITC analysis. After investigating a series of backbone sites, we found that CBA15-G3S with PS modification between the third G and fourth G of CBA15 showed higher binding affinity to Cd^2+^, with K_d_s of 46.6 ± 12.7 nM (Figure 4A), while the other PS-modified aptamers had K_d_s ranging from 105 nM to 389 nM (Table S2). Compared with CBA15 without PS modification (Figure 4B, K_d_ = 216.0 ± 43.3 nM), CBA15-G3S had about four times higher affinity than CBA15 to Cd^2+^. The results showed that PS modification at a favorable backbone site on the aptamer indeed greatly enhanced the aptamer affinity. An aptamer probe with higher affinity that can generate larger FA signal change upon Cd^2+^ binding is preferred for sensitive detection of Cd^2+^. Thus, we introduced TMR at the 12th T of CBA15-G3S to obtain a CBA15-G3S-T12-TMR probe, and applied it for FA detection of Cd^2+^.

### 3.3. Optimization of Aptamer FA Sensor for Cd^2+^

**Figure 5:**
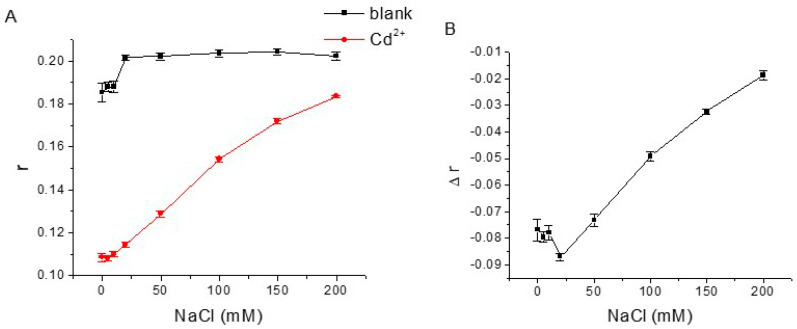
(**A**) Effects of NaCl concentration on FA responses of blank sample solution and the solution containing 1000 nM Cd^2+^ with 20 nM CBA15-G3S-T12-TMR. (**B**) The relationship between FA changes (Δr) caused by Cd^2+^ and NaCl concentration.

To achieve better performance of the aptamer FA sensor for Cd^2+^, we investigated the influence of NaCl concentration, MgCl_2_ concentration, and pH of binding buffer on FA responses of CBA15-G3S-T12-TMR to Cd^2+^. We first tested the effect of NaCl in binding buffer (Figure 5). In the absence of Cd^2+^, the FA value of the blank sample slowly increased with the addition of NaCl. When Cd^2+^ was present, the FA value sharply increased from 0.109 to 0.184 with the increase of NaCl. The FA change (Δr) showed a high value at 20 mM NaCl. With the increase of NaCl from 20 mM to 200 mM, the FA change gradually became smaller. The results indicate that a high ionic strength of buffer is unfavorable for Cd^2+^ binding with aptamers. Therefore, we chose to use 20 mM NaCl in binding buffer for subsequent experiments.

We further tested the influence of MgCl_2_ concentration in the binding buffer. As displayed in Figure S1, with the addition of MgCl_2_ from 0 to 5 mM, the FA value of the blank sample decreased from 0.197 to 0.174, and then changed slowly when the concentration of MgCl_2_ was higher than 5 mM. In the presence of Cd^2+^, the FA responses of CBA15-G3S-T12-TMR significantly increased with the addition of MgCl_2_. Figure S1B shows that the addition of MgCl_2_ caused decreased FA change (Δr), suggesting that MgCl_2_ was not necessary for larger FA change. Thus, the binding buffer with 20 mM NaCl was preferred for the subsequent investigations.

We also assessed the influence of the pH of binding buffer solution on the FA responses of CBA15-G3S-T12-TMR (Figure S2). When the pH was lower than 7.5, a relatively small FA change caused by Cd^2+^ was observed. In order to obtain a larger FA change, binding buffer solution at pH 7.5 was chosen for our FA assay.

### 3.4. Detection of Cd^2+^ with Aptamer FA Sensor

**Figure 6:**
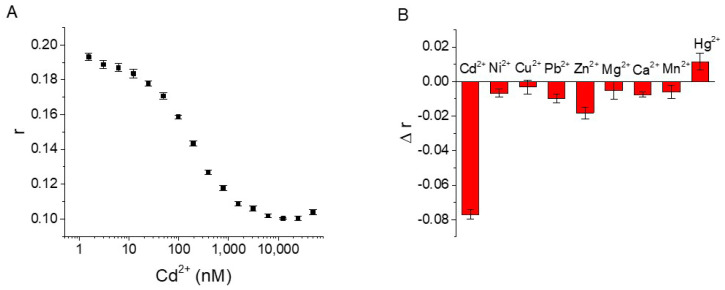
(**A**) FA detection of Cd^2+^ using CBA15-G3S-T12-TMR. (**B**) Selectivity test of the FA sensor using CBA15-G3S-T12-TMR for Cd^2+^ detection. A 20 nM aptamer probe was used, and the concentrations of tested metal ions were 1000 nM.

Under the optimized experimental conditions, we successfully achieved detection of Cd^2+^ by FA analysis with the aptamer probe CBA15-G3S-T12-TMR. As shown in Figure 6A, the FA value of the probe gradually decreased with the addition of Cd^2+^. The dynamic detection range was from 6.1 nM to 6250 nM, covering about three orders of magnitude. The nonlinear fitting equation of the response of FA to different concentrations of Cd^2+^ was obtained by GraphPad Prism software as y = (−0.0918 ± 0.0011)x/((171.8 ± 9.362) + x) + (0.1913 ± 0.0007) (R^2^ = 0.9987). The detection limit of Cd^2+^ was 6.1 nM (S/N = 3). This method meets the requirements for assessing Cd^2+^ in drinking water as set by the U.S. Environmental Protection Agency (EPA) (5.0 μg/L, corresponding to 44 nM). For comparison, we also tested the FA response of the aptamer without PS modification (CBA15-T12-TMR) (Figure S3). From the FA response curves, we estimated that the K_d_ values of CBA15-G3S-T12-TMR and CBA15-T12-TMR were 147.4 ± 6.7 nM (R^2^ = 0.9973) and 880.4 ± 25.5 nM (R^2^ = 0.9988), respectively, by non-linear fitting with GraphPad Prism software [28]. The detection limit of CBA15-T12-TMR to Cd^2+^ was determined to be 24.4 nM (S/N = 3). The results indicated that introduction of PS modification at the specific site of the aptamer greatly enhanced the binding affinity of the aptamer, and improved its FA sensitivity to Cd^2+^, while the aptamer CBA15-G3S-T12-TMR enabled a lower detection limit. Compared with other aptamer-based methods for Cd^2+^ detection [15,16,18,21,32,33,34], our method had better or comparable sensitivity (Table S3). Although the detection limit of our FA sensor was higher than that of the reported method [35], our strategy is simple, rapid, and requires only one TMR-labeled aptamer. In our method, quantification of Cd^2+^ can be achieved by mixing the TMR-labeled aptamer and Cd^2+^ solution, without need for separation and immobilization.

### 3.5. Selectivity Test and Practical Sample Analysis

We further tested the selectivity of the FA sensor for Cd^2+^ detection using CBA15-G3S-T12-TMR. As displayed in Figure 6B, other divalent metal ions including Cu^2+^, Ni^2+^, Pb^2+^, Zn^2+^, Hg^2+^, Mn^2+^, Mg^2+^, and Ca^2+^ did not cause significant FA changes. The results showed that our assay was selective for Cd^2+^ detection.

To explore whether this FA sensor is applicable to real samples, we detected Cd^2+^ spiked in complex sample matrix with binding buffer. As displayed in Figure S4, the FA responses of CBA15-G3S-T12-TMR in 20-fold diluted tap water and 20-fold diluted lake water showed almost similar performances with binding buffer. The detection limits of Cd^2+^ in real water samples were 6.1 nM. Cd^2+^ spiked in 20-fold diluted lake water and 20-fold diluted tap water exhibited good recoveries of 89.1–121.3% and 81.7–127.1%, respectively (Table S4). The results confirm that our aptamer FA sensor can be used for practical sample analysis.

## 4. Conclusions

In summary, we have reported a simple aptamer fluorescence anisotropy sensor for detection of Cd^2+^ using a TMR-labeled high-affinity aptamer with specific PS modification at the backbone. After screening different labeling sites on the aptamer, we identified the aptamer probe with a TMR labeled on the 12th T base of a 15-mer DNA aptamer (CBA15) that showed large FA signal change in response to Cd^2+^ binding. We also demonstrated that introduction of PS modification at the specific backbone site (the third G) of CBA15 greatly enhanced the binding affinity of the aptamer, with approximately four-fold improvement. The TMR-labeled aptamer probe with PS modification allowed sensitive selective detection of Cd^2+^, and the detection limit reached 6.1 nM Cd^2+^. Our aptamer FA sensor provides a direct method for Cd^2+^ detection without need for competition, separation, and immobilization. This FA method offers the advantages of simplicity, sensitivity, robustness, rapidity, and high throughput, showing the potential for analysis of Cd^2+^ in various applications, especially for fast onsite detection of Cd^2+^. This aptamer FA sensor circumvents the limitations of antibody-based FA assays for Cd^2+^ and certain methods using expensive instruments (e.g., ICP-MS). The introduction of PS modification provides an effective way to improve the affinity of aptamers for Cd^2+^, and the aptamers with higher affinity will have wide applications in developing biosensors. This strategy will be helpful for the affinity enhancement of aptamers for use with other targets.

## Figures and Tables

**Table 1:** List of the DNA oligonucleotides.

Name	Sequences
CBA15	5′-CGG GTT CAC AGT CCG-3′
CBA15-3′-TMR	5′-CGG GTT CAC AGT CCG-TMR-**3′** ^a^
CBA15-T5-TMR	5′-CGG G**T**(TMR)T CAC AGT CCG-3′
CBA15-T6-TMR	5′-CGG GT**T**(TMR) CAC AGT CCG-3′
CBA15-T12-TMR	5′-CGG GTT CAC AG**T**(TMR) CCG-3′
CBA15-5′-TMR	**5′**-TMR-CGG GTT CAC AGT CCG-3′
CBA15-G3S-T12-TMR	5′-CGG_PS_ GTT CAC AG**T**(TMR) CCG-3′ ^b^

^a^ The labeling sites of TMR are shown in bold. ^b^ The labeling site of PS modification in the backbone is shown with PS, indicating that the backbone between G3 and G4 had a PS modification.

## Data Availability

Not applicable.

## Supplementary Materials

The following supporting information can be downloaded at: https://www.mdpi.com/article/10.3390/bios12100887/s1, Table S1. List of the anti-Cd^2+^ aptamer sequences with PS modification at different labeling sites. Table S2. Binding affinity of PS modified aptamers and unlabeled aptamer characterized by ITC. Table S3. Comparison of some aptamer based methods for Cd^2+^ detection. Table S4 Detection of Cd^2+^ spiked in complex sample matrix. Figure S1. (A) Effect of MgCl_2_ concentration on FA responses of CBA15-G3S-T12-TMR in the absence or in the presence of Cd^2+^. (B) The relationship between FA changes (Δr) and MgCl_2_ concentration. Figure S2. (A) Effect of pH of binding buffer on FA responses of CBA15-G3S-T12-TMR in the absence or in the presence of Cd^2+^. (B) The FA changes (Δr) caused by Cd^2+^ at various pH of the binding buffer. Figure S3. Comparison of FA detection Cd^2+^ with CBA15-G3S-T12-TMR and CBA15-T12-TMR. Figure S4. Detection of Cd^2+^ in the binding buffer, 20-fold diluted tap water or 20-fold diluted lake water with the aptamer FA sensor by using CBA15-G3S-T12-TMR.
