# Supplementary material for: A Sensitive Aptamer Fluorescence Anisotropy Sensor for Cd2+ Using Affinity-Enhanced Aptamers with Phosphorothioate Modification
Source: Biosensors (Basel). 2022 Oct 17;12(10):887. doi: 10.3390/bios12100887 (PMC9599812; doi:10.3390/bios12100887)
Supplement: Supplementary file 1 [file biosensors-12-00887-s001.zip › biosensors-1947676-supplementary.pdf]

biosensors

Supporting Information

A Sensitive Aptamer Fluorescence Anisotropy Sensor for Cd^{2+} Using Affinity-Enhanced Aptamers with Phosphorothioate Modification

Hao Yu [1,2] and Qiang Zhao [1,2,3,*]

[1] State Key Laboratory of Environmental Chemistry and Ecotoxicology, Research Center for Eco-Environmental Sciences, Chinese Academy of Sciences, Beijing 100085, China
[2] University of Chinese Academy of Sciences, Beijing 100049, China
[3] School of Environment, Hangzhou Institute for Advanced Study, University of Chinese Academy of Sciences, Hangzhou 310000, China
* Correspondence: qiangzhao@rcees.ac.cn

Table S1. List of the anti-Cd^{2+} aptamer sequences with PS modification at different labeling sites.

Name	Sequences
CBA15-C1S	5'-C$_{PS}$GG GTT CAC AGT CCG-3'
CBA15-G2S	5'-CG$_{PS}$G GTT CAC AGT CCG-3'
CBA15-G3S	5'-CGG$_{PS}$ GTT CAC AGT CCG-3'
CBA15-G4S	5'-CGG G$_{PS}$TT CAC AGT CCG-3'
CBA15-T5S	5'-CGG GT$_{PS}$T CAC AGT CCG-3'
CBA15-T6S	5'-CGG GTT$_{PS}$ CAC AGT CCG-3'
CBA15-C7S	5'-CGG GTT C$_{PS}$AC AGT CCG-3'
CBA15-A8S	5'-CGG GTT CA$_{PS}$C AGT CCG-3'
CBA15-C9S	5'-CGG GTT CAC$_{PS}$ AGT CCG-3'
CBA15-A10S	5'-CGG GTT CAC A$_{PS}$GT CCG-3'
CBA15-G11S	5'-CGG GTT CAC AG$_{PS}$T CCG-3'
CBA15-T12S	5'-CGG GTT CAC AGT$_{PS}$ CCG-3'
CBA15-C13S	5'-CGG GTT CAC AGT C$_{PS}$CG-3'
CBA15-C14S	5'-CGG GTT CAC AGT CC$_{PS}$G-3'

Table S2. Binding affinity of PS modified aptamers and unlabeled aptamer characterized by ITC.

Aptamer	Kd/nM	ΔH (kJ/mol)	$-T\Delta S$ (kJ/mol)
CBA15	216 ± 43.3	–71.6 ± 1.67	33.5
CBA15-C1S	148 ± 13.7	–70.6 ± 0.7	31.6
CBA15-G2S	176 ± 19.7	–70.6 ± 0.9	32.0
CBA15-G3S	46.6 ± 12.7	–75.0 ± 1.5	33.1
CBA15-G4S	389 ± 22.9	–79.2 ± 0.7	42.6
CBA15-T5S	169 ± 29.3	–74.9 ± 1.5	36.2
CBA15-T6S	261 ± 35.5	–70.5 ± 1.2	32.8
CBA15-C7S	368 ± 38.0	–59.3 ± 0.9	22.5
CBA15-A8S	182 ± 28.1	–74.3 ± 1.3	35.8
CBA15-C9S	268 ± 25.8	–80.1 ± 1.0	42.6
CBA15-A10S	105 ± 11.5	–75.2 ± 0.8	35.3
CBA15-G11S	177 ± 50.4	–72.0 ± 2.3	33.4
CBA15-T12S	360 ± 63.2	–85.9 ± 2.1	49.1
CBA15-C13S	266 ± 26.9	–79.5 ± 1.1	41.9
CBA15-C14S	200 ± 57.9	–67.2 ± 2.4	29.0

Table S3. Comparison of some aptamer based methods for Cd^{2+} detection.

Methods	Detection Limit (nM)	Detection Range (nM)	Application	Ref.
Aptamer fluorescence assay based on conformational switching of SYBR green I and probe	3.0	10.0 – 2000	River, pond water, tap water and mine pit water	32
A fluorescence quenching sensor	2.2	7.2 – 5000	River, pond water, mine pit water, tap water and blood samples	33
A light-up fluorescence biosensor	40.0	0 – 1000	Lake water	15
Aptamer colorimetric assay using cationic polymer and gold nanoparticles	4.6	10.0 – 4000	Not mentioned	16
Fluorescence assay using MOPS to amplify signal	17.1	44.5 – 3.6×10^4	River and ultrapure water	20
A phosphorescence resonance energy transfer aptasensor	3.1	4.4 – 444.8	Bottle water and yesso scallops	34
A ratiometric electrochemical sensor	6.2	17.8 – 7116.8	Real mussel samples	18
Colorimetric assay using G-quadruplex three-way junction	0.01	0.01 – 1000	River water samples	35
FA sensor using TMR labeled aptamer	6.1	6.1 – 6250	Lake water and tap water	This work

MOPS: 3-(N-morpholino)propane sulfonic acid.

Table S4. Detection of Cd^{2+} spiked in complex sample matrix.

Complex samples	Cd^{2+} Spiked (nM)	Cd^{2+} Found (nM)	Recoveries (%)
Lake water (20-fold diluted)	24	29.1 ± 2.1	121.3 ± 8.7
	49	48.9 ± 4.9	99.7 ± 10.0
	98	107.6 ± 9.5	109.8 ± 10.0
	391	378.1 ± 16.2	96.7 ± 4.1
	781	695.6 ± 62.5	89.1 ± 8.0
Tap water (20-fold diluted)	24	30.5 ± 4.7	127.1 ± 19.5
	49	52.8 ± 3.5	107.7 ± 7.2
	98	100.9 ± 3.5	103.0 ± 3.6
	391	358.7 ± 15.0	91.8 ± 3.8
	781	638.4 ± 41.6	81.7 ± 5.3

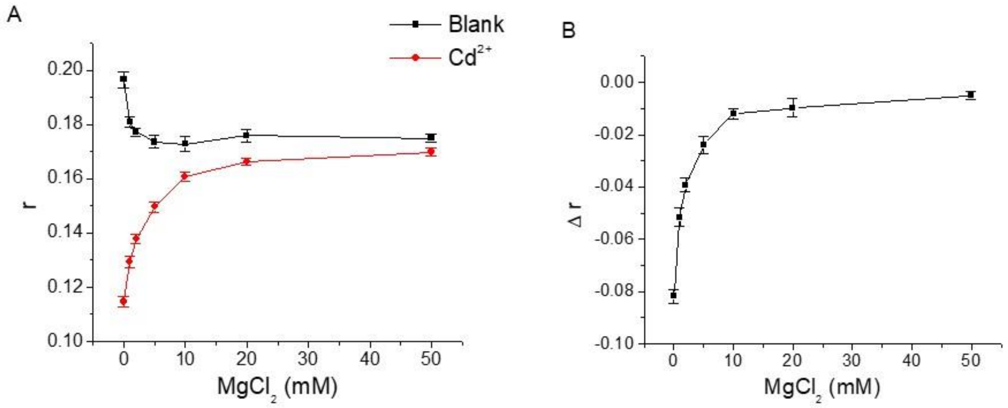

Figure S1. (**A**) Effect of $MgCl_2$ concentration on FA responses of CBA15-G3S-T12-TMR (20 nM) in the absence or in the presence of Cd^{2+} (1000 nM). (**B**) The relationship between FA changes (Δr) and $MgCl_2$ concentration. The binding buffer contained 20 mM Tris-HCl (pH 7.5), 20 mM NaCl and varying concentrations of $MgCl_2$.

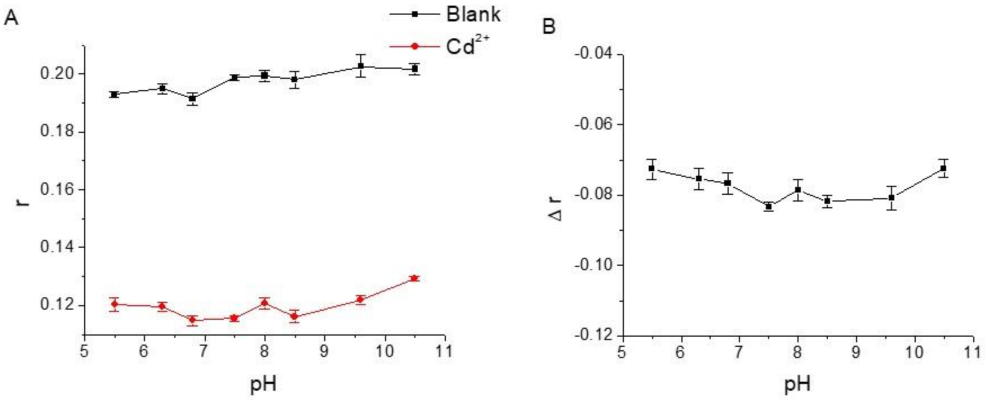

Figure S2. (**A**) Effect of pH of binding buffer (5.5, 6.3, 6.8, 7.5, 8.0, 8.5, 9.6 and 10.5) on FA responses of CBA15-G3S-T12-TMR (20 nM) in the absence or in the presence of Cd^{2+} (1000 nM). (**B**) The FA changes (Δr) caused by Cd^{2+} at various pH of the binding buffer. The binding buffer contained 20 mM Tris-HCl and 20 mM NaCl.

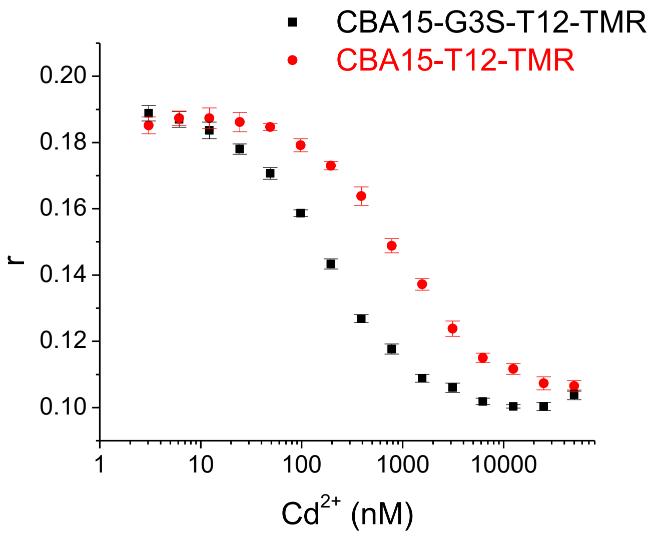

Figure S3. Comparison of FA detection Cd^{2+} with CBA15-G3S-T12-TMR and CBA15-T12-TMR.

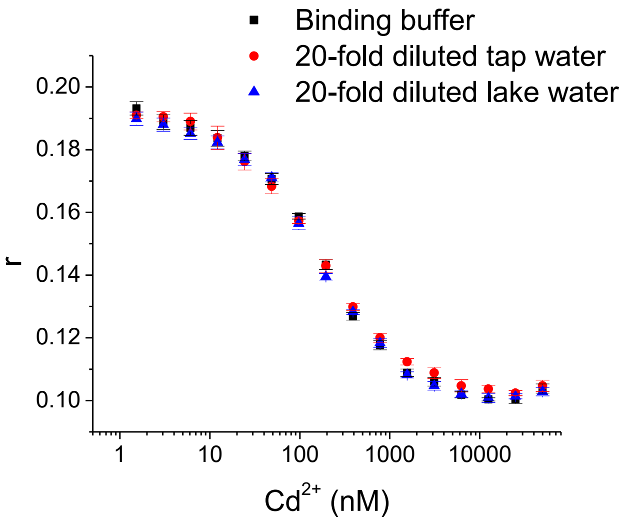

Figure S4. Detection of Cd^{2+} in the binding buffer, 20-fold diluted tap water or 20-fold diluted lake water with the aptamer FA sensor by using CBA15-G3S-T12-TMR.
